# Venetoclax combined with azacitidine in elderly acute myeloid leukemia: A retrospective comparison of 14-day vs 28-day dosing regimens

**DOI:** 10.1097/MD.0000000000043979

**Published:** 2025-08-15

**Authors:** Zhuruohan Yu, Shuangyue Li, Renzhi Pei, Ying Lu, Yuxiao Wang, Jiaojiao Yuan

**Affiliations:** a Department of Hematology, The Affiliated People’s Hospital of Ningbo University, Ningbo, China; b Institute of Hematology, Ningbo University, Ningbo, China.

**Keywords:** acute myeloid leukemia, efficacy, elderly, myelosuppression, short-term, venetoclax

## Abstract

However, this study has several limitations that must be acknowledged. First, the non-randomized allocation of treatment duration introduces potential selection bias, particularly as frailer patients were more likely to receive shorter therapeutic cycles, which may have confounded outcome assessments. Background: Although the standard 28-day venetoclax (VEN) regimen combined with azacitidine (AZA) improves outcomes in elderly patients with acute myeloid leukemia, emerging evidence suggests that shorter VEN cycles may maintain efficacy with enhanced safety. We retrospectively analyzed 90 treatment-naive elderly patients with acute myeloid leukemia receiving VEN + AZA (VA): 47 patients (14-day VEN) and 43 patients (28-day VEN). The outcomes included clinical remission rates, hematologic recovery, adverse events, and survival metrics. Both groups achieved comparable clinical remission rates (CRc: 57.4% vs 58.1%, *P* = .947). The 14-day cohort demonstrated significantly faster neutrophil recovery (median 12.5 vs 26 days, *P* < .01) and reduced febrile neutropenia (73.3% vs 90.9%, *P* < .05), with trends toward fewer grade ≥3 infections. At a median follow-up of 494 days, no significant differences in median overall survival (OS: 494 vs 578 days, HR = 1.17, 95%CI 0.64–2.14) or event-free survival (416 vs 454 days, HR = 1.09, 95%CI 0.61–1.96) were observed. A 14-day VA regimen showed antileukemic efficacy comparable to the 28-day protocol while mitigating myelosuppressive sequelae. This abbreviated approach may optimize tolerability in frail elderly patients who are ineligible for prolonged low-intensity chemotherapy. Prospective validation is warranted to refine risk-adapted dosing strategies.

## 1. Introduction

Acute myeloid leukemia (AML), a clinically and molecularly heterogeneous hematologic malignancy, demonstrates an age-dependent incidence profile with a median diagnosis age of 68 years.^[1,2]^ Elderly AML patients frequently exhibit compromised performance status, increased comorbidities, and higher frequencies of high-risk cytogenetic or molecular abnormalities, collectively contributing to dismal outcomes.^[3,4]^ While intensive chemotherapy remains standard for younger cohorts, older patients face limited therapeutic options, reflected in 5-year overall survival (OS) rates below 20% beyond the age of 60.^[5]^

Venetoclax (VEN), a selective B-cell lymphoma 2 (BCL-2) inhibitor combined with AZA, has emerged as a frontline therapy for AML patients unfit for intensive chemotherapy.^[6]^ Current guidelines recommend 28-day VEN cycles (400 mg/day following a 3-day ramp-up) to balance efficacy and toxicity.^[7,8]^ However, prolonged myelosuppression remains a critical limitation; the landmark VIALE-A trial reported 24% permanent VEN discontinuations due to adverse events, predominantly febrile neutropenia, with 53% requiring dose interruptions.^[9]^ These challenges are amplified in real-world practice, where frailty and comorbidities often preclude tolerance to extended VEN exposure.

Given the unmet need for regimens minimizing toxicity without compromising efficacy in vulnerable populations, this study evaluates whether shortening VEN duration to 14 days preserves antileukemic activity while reducing treatment-related morbidity in Chinese elderly AML patients.

## 2. Patients and methods

### 2.1. Patients

This study included AML (non-M3) patients aged ≥60 years who were treated at the Department of Hematology of Ningbo University Affiliated People’s Hospital from January 2019 to December 2023. All patients underwent a comprehensive diagnostic workup, including morphological, immunologic, cytogenetic, and molecular biological testing, and were diagnosed according to the 2022 World Health Organization AML diagnostic criteria.^[10]^ They were risk-stratified based on the 2022 European LeukemiaNet (ELN) criteria.^[11]^ The study protocol was approved by the Institutional Review Board of Ningbo University Affiliated People’s Hospital and conducted under the Declaration of Helsinki.

### 2.2. Treatment

Patients were allocated to 14-day (short-term) or 28-day (long-term) VEN regimens based on clinician evaluation of frailty, organ dysfunction, disease risk, and tolerance. VEN was administered with a standardized ramp-up protocol: 100 mg orally on day 1, 200 mg on day 2, and 400 mg on day 4, maintained through day 14 or 28 for respective cohorts. AZA (75 mg/m^2^) was delivered subcutaneously/intravenously on days 1 to 7. Dose modifications mandated 50% VEN reduction (≤200 mg/day) with moderate Cytochrome P450 3A (CYP3A) inhibitors and further adjustment to 100 mg/day with potent inhibitors. Dose reductions or treatment delays were implemented for protocol-defined intolerable toxicities. All patients received tumor lysis syndrome prophylaxis and supportive transfusions during myelosuppression. Post-remission consolidation continued the VA regimen, with transplantation eligibility determined by ELN 2022 risk stratification. nonresponders after 2 cycles were referred for clinical trials or alternative regimens.

### 2.3. Efficacy assessment

The efficacy of AML treatment was evaluated using ELN response criteria,^[10]^ which included complete remission (CR), complete remission with incomplete hematologic recovery (CRi), and partial remission. The combined complete cytogenetic remission (CRc) and overall remission (ORR) were calculated as the sum of CR and CRi, and the sum of CR, CRi, and partial remission, respectively. Minimal residual disease (MRD) was assessed by immunophenotyping of bone marrow cells using 4-color immunolabeled flow cytometry with a threshold of 0.1% for MRD determination. Neutrophil and platelet recovery times were defined as the number of days after chemotherapy when the neutrophil count exceeded 0.5 × 10^9^/L and the platelet count exceeded 20 × 10^9^/L, respectively. Adverse events were graded according to the National Cancer Institute Common Terminology Criteria for Adverse Events (version 4.0). Overall survival (OS) and event-free survival (EFS) were calculated from the start of diagnosis and treatment to endpoints, such as death or recurrence.

### 2.4. Statistical methods

Statistical analyses were performed using the SPSS software (version 26.0, Chicago). Categorical variables were compared using Pearson chi-squared test, with Fisher exact test substituted when the total sample size was < 40 or the expected cell frequency was <1. Continuous variables conforming to a normal distribution were analyzed using an independent-sample *t*-test. The Kaplan–Meier method was used to estimate survival outcomes (OS and EFS) and compared using the log-rank test. Statistical significance was set at a 2-tailed *P*-value <.05.

## 3. Study results

### 3.1. Patient demographics

This study enrolled 90 elderly patients with newly diagnosed AML, stratified into short-term (n = 47) and long-term (n = 43) treatment groups. Baseline characteristics included a median age of 70 years (range 60–87), Eastern Cooperative Oncology Group performance status 2 (0–3), and bone marrow blast count of 45.5% (20%–92.5%). Prognostic stratification revealed 7.8% favorable, 27.8% intermediate, and 64.4% adverse risks. Key genetic features showed a complex karyotype predominance (27.7% vs 23.3% between groups), and DNMT3A was the most frequent molecular alteration (23.4% vs 32.6%). All patients received a median of 2 courses of the VA regimen. The detailed demographic and clinicopathological characteristics are presented in Table 1.

**Table 1:** Patients characteristics in the short-term group and the long-term group.

Variable	Short-term group (N = 47)	Long-term group (N = 43)	*P*-value
Age, year, median (range)	71 (60–87)	67 (60–81)	.533
≤70	23 (48.9)	25 (58.1)	
>70	24 (51.1)	18 (41.9)	
Sex, n (%)			
Male	26 (55.3)	19 (44.2)	.291
Female	21 (44.7)	24 (55.8)	
Classification, n (%)			
Primary	37 (78.7)	35 (81.4)	.752
Secondary	10 (21.3)	8 (18.6)	
ECOG score, n (%)			
0–1 points	22 (46.8)	25 (58.2)	.743
2–4 points	25 (53.2)	18 (41.8)	
Risk classification, n (%)			
Favorable	3 (6.4)	4 (9.3)	.108
Intermediate	9 (19.1)	16 (37.2)	
Adverse	35 (74.5)	23 (53.5)	
Cytogenetic mutations, n (%)			
Complex karyotype	13 (27.7)	10 (23.3)	.632
Seven or 7q deletion	3 (6.4)	1 (2.3)	.618
Five or 5q deletion	5 (10.6)	2 (4.7)	.438
Molecular biology mutations			
NPM1	8 (17.0)	6 (14.0)	.688
DNMT3A	11 (23.4)	14 (32.6)	.356
CEBPA	3 (6.4)	6 (14.0)	.301
FLT3-ITD	6 (12.8)	5 (11.6)	.869
FLT3-TKD	1 (2.1)	5 (11.6)	.100
ASXL1	6 (12.8)	4 (9.3)	.742
IDH1	4 (8.5)	6 (14.0)	.510
IDH2	4 (8.5)	4 (9.3)	1.000
PTPN11	3 (6.4)	3 (7.0)	1.000
TP53	7 (14.9)	5 (11.6)	.649
Bone marrow blast cell rate, median (range)	40.5 (16–88)	52 (20–92.5)	.345
Blood count during diagnosis, median (range)			
White blood cell count, ×10^9^/L	4.9 (0.4–152.7)	4.4 (0.5–257)	.345
Neutrophils count, ×10^9^/L	1.1 (0.08–132.9)	0.78 (0.04–121.9)	.303
Hemoglobin, g/L	78.5 (44–132)	80 (35–129)	.365
Platelet count, ×10^9^/L	50 (7–704)	55 (4–402)	.358
Treatment cycles for VA regimen, median (range)	2 (1–10)	2 (1–8)	.594

ECOG = Eastern Cooperative Oncology Group, VA = venetoclax combined with azacitidine.

### 3.2. Response to treatment

The short-term group achieved comparable CRc rates to long-term therapy after single-course induction (55.3% vs 53.5%), with superior performance in elderly patients aged ≥ 70 years (CRc 54.2% vs 38.9%). Notably, the short-term group maintained a competitive ORR (82.9% vs 81.4%) and showed a particular advantage in the adverse-risk subgroup (CRc 51.4% vs 47.8%).

The MRD negativity rates improved substantially in both arms with treatment consolidation (short-term: 61.5%; long-term: 73.9%), although the short-term protocol required fewer median treatment courses (2 vs 2, range 1–10 vs 1–6). Strikingly, the short-term approach achieved 14.3% higher CRc rates than long-term therapy in the elderly cohort (≥70 years) despite comparable baseline characteristics.

Consolidation strategies included hematopoietic stem cell transplantation in 11.1% of the responders, with the short-term group demonstrating 75% utilization of allogeneic hematopoietic stem cell transplantation (3/4) versus 83% (5/6) in the long-term group. The complete therapeutic responses are detailed in Table 2.

**Table 2:** Analysis of therapeutic effects in the short-term group and the long-term group.

	Short-term group (N = 47)	Long-term group (N = 43)	*P*-value
CRc rate, n (%)			
One course of treatment	26 (55.3)	23 (53.5)	.862
Two or more courses of treatment	27 (57.4)	25 (58.1)	.947
ORR rate, n (%)			
One course of treatment	39 (82.9)	35 (81.4)	.844
Two or more courses of treatment	40 (85.1)	37 (86.0)	.899
MRD-negative rate, n (%)			
One course of treatment	11 (40.7)	14 (56.0)	.357
Two or more courses of treatment	16 (61.5)	17 (73.9)	.271
CRc rate based on age, n (%)			
≤70	13 (56.5)	16 (64.0)	.597
>70	13 (54.2)	7 (38.9)	.327
CRc rate based on risk classification, n (%)			
Favorable and intermediate	8 (66.7)	12 (60)	.659
Adverse	18 (51.4)	11 (47.8)	.778

CRc = complete cytogenetic remission, MRD = minimal residual disease, ORR = overall remission.

### 3.3. Safety analysis

No 30-day mortality occurred in either of the groups. At 60 days, the mortality rates tended to be lower in the short-term group (4.3% vs 9.3%, *P* = .42), with distinct mortality etiologies: renal/cardiac events (acute kidney failure, cardiogenic shock) in the short-term group versus infectious complications (severe pneumonia, septic shock) in the long-term group.

Hematological safety profiles significantly favored the short-term regimen, demonstrating lower rates of grade ≥ 3 toxicities: leukopenia (80.9% vs 95.3%, *P* = .036), neutropenia (78.7% vs 97.7%, *P* = .006), and thrombocytopenia (78.7% vs 95.3%, *P* = .020). The short-term group also showed reduced febrile neutropenia incidence (72.3% vs 90.7%, *P* = .026), requiring fewer VEN dose reductions (21.2% vs 56.3%), and achieving faster neutrophil recovery (median 12.5 vs 36 days, *P* = .001). Table 3 details the blood count recovery during the first course of chemotherapy.

**Table 3:** Adverse events during the first course of chemotherapy in the short-term group and the long-term group.

	Short-term group (N = 47)	Long-term group (N = 43)	*P*-value
Hematology adverse events above level III, n (%)			
Leukopenia	38 (80.9)	41 (95.3)	.036
Neutropenia	37 (78.7)	42 (97.7)	.006
Hemoglobin reduction	34 (72.3)	32 (74.4)	.824
Thrombocytopenia	37 (78.7)	41 (95.3)	.020
Non hematology adverse events above level III, n (%)			
Febrile neutropenia	34 (72.3)	39 (90.7)	.026
Infect	34 (72.3)	36 (83.7)	.195
Hemorrhage	12 (25.5)	16 (37.2)	.232
Cardiac insufficiency	6 (12.8)	8 (18.6)	.445
Hepatic insufficiency	6 (12.8)	8 (18.6)	.445
Renal insufficiency	7 (14.9)	9 (20.9)	.454
Dizziness and fatigue	23 (48.9)	20 (60.6)	.303
Nausea and vomiting	11 (23.4)	11 (25.6)	.810
Tumor lysis syndrome	2 (4.3)	3 (7.0)	.537

Non-hematologic adverse events showed comparable frequencies between the groups, including invasive fungal infections (6.4% vs 9.3%, *P* = .705) and organ dysfunction parameters. Prophylactic antifungal use was similar across the treatment arms (27.7% vs 32.6%). The comprehensive safety profiles are presented in Table 4.

**Table 4:** Recovery time of blood count during the first course of chemotherapy in the short-term group and the long-term group.

	Short-term group (N = 47)	Long-term group (N = 43)	*P*-value
Hematology adverse events above level III, n (%)			
Leukopenia	38 (80.9)	41 (95.3)	.036
Neutropenia	37 (78.7)	42 (97.7)	.006
Anemia	34 (72.3)	32 (74.4)	.824
Thrombocytopenia	37 (78.7)	41 (95.3)	.020
Non hematology adverse events above level III, n (%)			
Febrile neutropenia	34 (72.3)	39 (90.7)	.026
Infect	34 (72.3)	36 (83.7)	.195
Hemorrhage	12 (25.5)	16 (37.2)	.232
Cardiac insufficiency	6 (12.8)	8 (18.6)	.445
Hepatic insufficiency	6 (12.8)	8 (18.6)	.445
Renal insufficiency	7 (14.9)	9 (20.9)	.454
Dizziness and fatigue	23 (48.9)	20 (60.6)	.303
Nausea and vomiting	11 (23.4)	11 (25.6)	.810
Tumor lysis syndrome	2 (4.3)	3 (7.0)	.537

### 3.4. Survival analysis

With a median follow-up of 697 days (range: 37–1170) and 3 patients lost to follow-up, mortality rates were comparable between groups: 47.8% (22/46) in the short-term cohort versus 48.8% (20/41) in the long-term cohort. Infection-related deaths predominated in both groups (short-term, 9/22; long-term, 12/20), with disease progression being the second leading cause.

Posttransplant outcomes showed sustained remission in 3 short-term patients (one recurrence) versus 4 long-term survivors (one recurrence, 3 remissions). Four long-term posttransplant deaths occurred, which were attributed to multidrug-resistant sepsis (n = 1), severe pneumonia, and disease progression.

**Figure 1.**
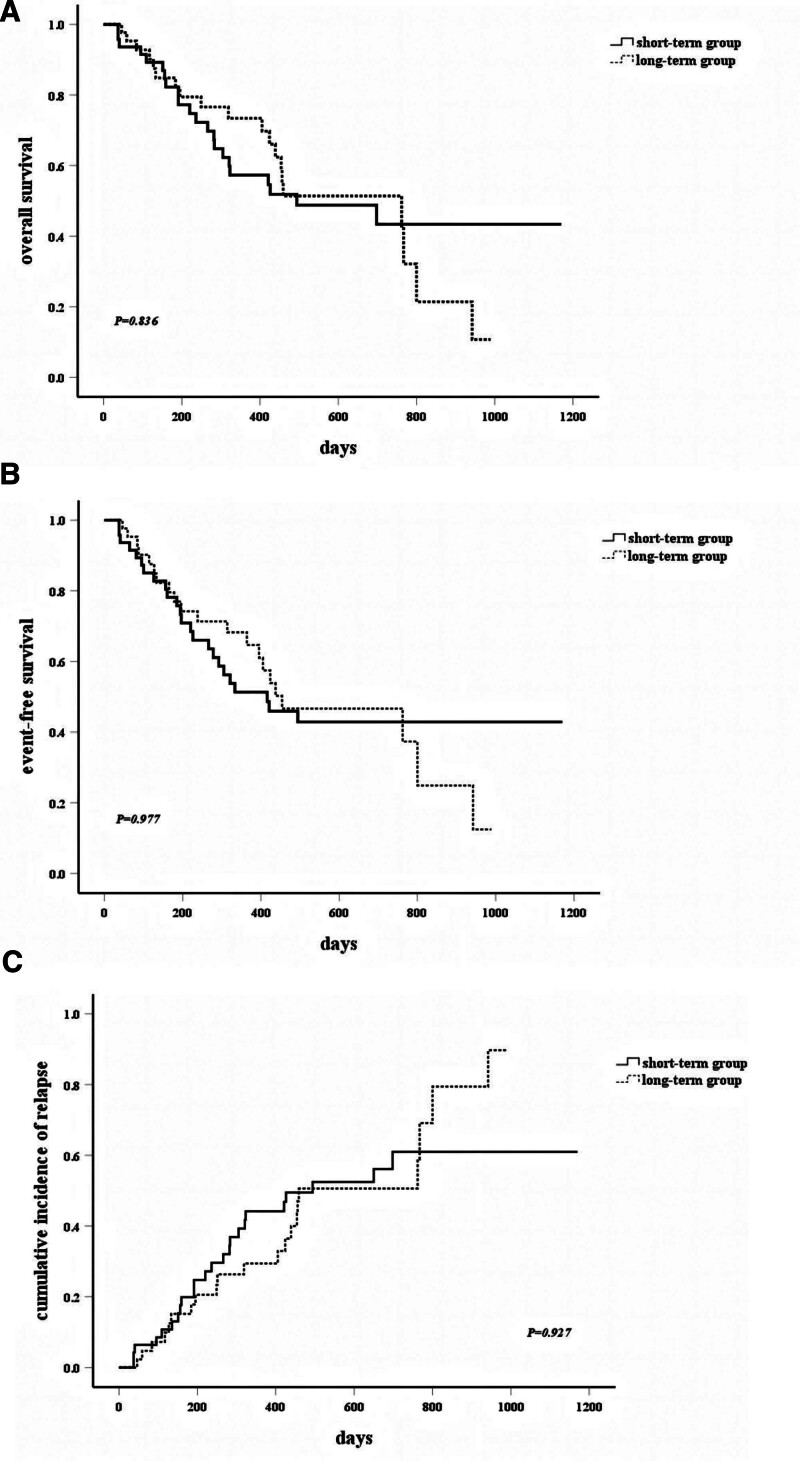
(A) Overall survival of the 2 groups. (B) EFS of the 2 groups. (C) CIR of the 2 groups. CIR = cumulative incidence of relapse, EFS = event-free survival.

**Figure 2.**
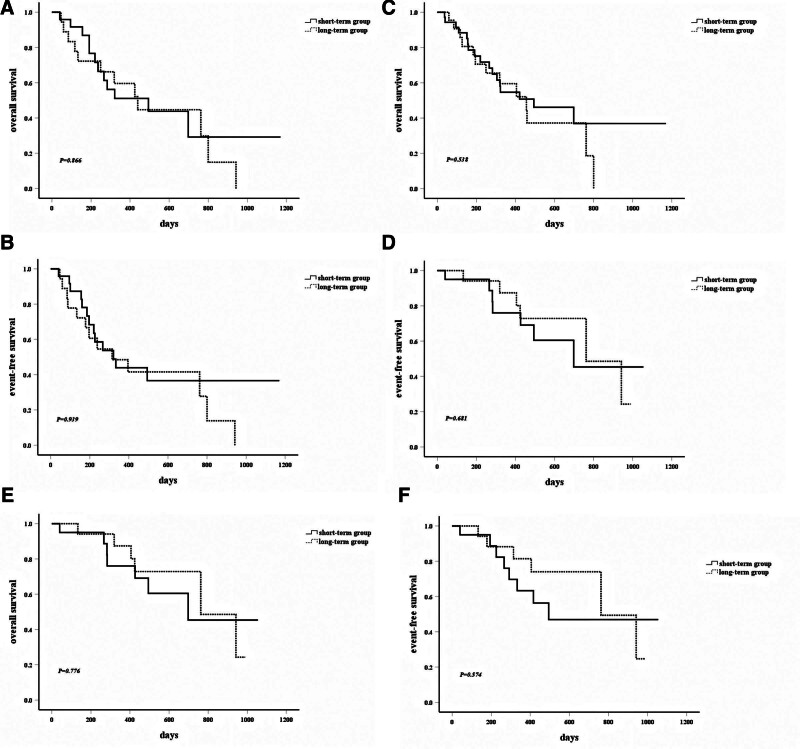
(A) Overall survival of the 2 groups of patients aged >70 years. (B) EFS of the 2 groups of patients aged >70 years. (C) OS of the 2 groups of patients with adverse-risk stratification. (D) EFS of the 2 groups of patients with adverse-risk stratification. (E) OS of the 2 groups of patients with MRD-negative. (F) EFS of the 2 groups of patients with MRD-negative. EFS = event-free survival, MRD = minimal residual disease, OS = overall survival.

No statistically significant differences were observed in OS (median 494 vs 578 days) or EFS (median 416 vs 454 days) between the short- and long-term groups (Fig. 1A and B). Similarly, cumulative relapse incidence (Fig. 1C) and subgroup analyses stratified by age, risk status, or MRD status showed no significant disparities in OS/EFS (Fig. 2A–F).

## 4. Discussion

AML is an aggressive malignancy with a poor prognosis and a high recurrence rate. Age is an independent prognostic risk factor, and elderly patients have a worse prognosis than younger patients.^[12]^ Treating elderly patients with AML involves several risks and challenges. VEN, a selective BCL-2 inhibitor, has shown efficacy in AML^[13]^; however, monotherapy is prone to drug resistance.^[14]^ AZA enhances VEN’s antitumor effect by activating the transcription of the proapoptotic protein NOXA.^[15]^ The combination of VEN and AZA induces strong and long-lasting antileukemic effects by blocking the energy metabolism of leukemic stem cells. This regimen is recommended for elderly AML patients who are unsuitable for intensive chemotherapy. Studies have shown that the remission rate increases with VEN doses ≤ 400 mg/day, but higher doses do not significantly improve remission or increase adverse events.^[16]^ International guidelines recommend a daily dose of 400 mg when VEN is combined with hypomethylating agents (HMAs). VEN exposure is only affected by food and CYP3A modulators and is only higher in Asian subjects and those with severe liver injury; the duration of treatment is clear, but inconsistent with clinical practice.^[17]^

Willekens et al reported an ORR of 68.3% in 82 newly diagnosed AML patients treated with a 7 + 7 VA regimen, similar to the 7 + 28 regimen. The 7 + 7 regimen has advantages over the conventional 7 + 28 dose.^[18]^ Aiba et al found similar CRc and OS rates in the VEN14 and VEN28 groups, with the VEN14 group having a lower incidence of febrile neutropenia and shorter hospitalization time.^[19]^ In this study, the CRc rates were 57.4% and 58.1%, and the ORR rates were 85.1% and 86% in the short- and long-term groups, respectively. The remission rate was similar in both groups, with most patients achieving remission during the first course of therapy. The CRc rate in this study was lower than that in the VIALE-A trial (66.4%), possibly because of the inclusion of all elderly patients. For patients aged ≥70 years, the ORR in the short-term group was 93.33%, which was significantly higher than that reported in other studies and similar to that in the long-term group. The CRc rate in the short-term group (54.2%) was higher than that in the long-term group (38.9%).^[20]^

The short-term regimen alleviated treatment-related adverse events, especially myelosuppression, shortened the duration of neutropenia deficiency, and reduced the incidence of severe infections. DiNardo et al reported CR/CRi rates of up to 60% for VA regimens in elderly AML patients with a poor prognosis.^[21]^ In this study, the CRc rates were similar in both groups of patients with poor prognosis, suggesting that shortening the VEN use duration can achieve the same near-term efficacy of 28 days.

Regarding safety, a prospective study by the SEIFEM team showed a high incidence of infection complications in patients receiving HMAs + VEN.^[22]^ This study emphasizes the importance of the prevention of infection during HMAs + VEN treatment. VEN is a CYP3A4 inhibitor that significantly interacts with azole antifungal drugs and requires dose adjustment.^[23]^ Antimicrobial prophylaxis is recommended during AML treatment to reduce the infection risk, including invasive fungal infections (IFIs).^[24]^ In this study, 30% of the patients received prophylactic antifungal therapy. Due to azole drug use, 27.7% and 32.6% of patients in the short- and long-term groups, respectively, reduced their VEN dosage. The treatment course length had no significant effect on the dose adjustment. During the first induction course, 21.2% and 56.2% of patients in the short- and long-term groups, respectively, needed shortened therapy, primarily due to febrile neutropenia. Shortening VEN use has reduced the incidence of therapy by more than half. Prolonging VEN use increased myelosuppression severity. The incidence of grade III-IV neutropenia was 97.7% in the long-term group, which was significantly higher than that in the short-term group (78.7%; *P* = .006). This may affect the efficacy of therapy.

The incidence of other hematological adverse events, such as anemia and thrombocytopenia, was similar. Foreign phase III studies showed a lower incidence of grade III or higher hematologic adverse events with the VA regimen for elderly patients with AML than in this study. This difference may be due to a poorer physical status, more comorbidities, and poorer tolerance to the same course and dose of VEN among Chinese patients, leading to higher myelosuppression. The short-term group had a significantly shorter neutrophil recovery time (median 12.5 days), more than half that of the long-term group. The long-term group had the highest incidence of IFIs before CR due to more severe and prolonged neutropenia. The incidences of possible or confirmed IFIs in the short- and long-term groups were 6.4% and 9.3%, respectively. There is a correlation between the severity and duration of neutropenia and the incidence of IFIs in patients with hematologic malignancies.^[25]^

Common non-hematologic adverse events were febrile neutropenia, infection, dizziness, and fatigue, with a significantly lower incidence in the short-term group than in the long-term group (72.3% vs 90.7%, *P* = .026). A short-term VA regimen may significantly shorten the duration of neutropenia, reduce the incidence of infection, and potentially reduce IFIs. Other non-haematological adverse events, such as dizziness, fatigue, and vital organ insufficiency, may also be reduced by the short-term VA regimen. The incidence of tumor lysis syndrome was similar in both groups (4.3% vs 7.0%).

Several studies have shown that VA regimens can significantly improve survival and prolong survival in elderly patients with AML. Bouligny et al found that a reduced VEN regimen for 14 days resulted in significantly longer OS (8.9 months vs 2.1 months, *P *= .0002) and progression-free survival (8.2 months vs 1.5 months, *P* < .0001) compared to 28 days.^[26]^ Another retrospective analysis showed a median OS of 18.6, 21.3, and 13.2 months for different VEN courses (14, 21, and 28 days) combined with HMAs, respectively, with no significant difference.^[27]^ The OS and EFS in this study were similar to those reported in other studies. No significant differences in OS and EFS were observed between the 2 groups in patients with advanced age, poor prognosis, or MRD-negative status. However, shortening the VEN regimen in patients with poor cytogenetic and molecular biological factors may increase susceptibility to relapse. No significant difference in the cumulative incidence of relapse was found between the 2 groups, requiring confirmation from large-scale prospective studies. Elderly patients with AML are less tolerant of long-term regimens and have a high incidence of serious infections, making a short-course VA regimen more suitable. Although MRD negativity was associated with better OS and EFS,^[28]^ the total MRD negativity rate was similar in both groups, with no significant difference in survival time. In summary, shortening VEN use may not affect the long-term survival.

However, this study has several limitations that must be acknowledged. First, the non-randomized allocation of treatment duration introduces potential selection bias, particularly as frailer patients were more likely to receive shorter therapeutic cycles, which may have confounded outcome assessments. Second, while elderly AML patients exhibit increased prevalence of adverse cytogenetic and molecular profiles – including complex karyotypes and mutations in TP53 and DNMT3A – the limited subgroup sample sizes precluded meaningful stratification by genomic features. Third, the analysis did not account for potential confounding from allogeneic hematopoietic stem cell transplantation outcomes. Finally, the relatively small cohort size and median follow-up duration of 697 days restrict the statistical power to detect long-term survival differences. Future investigations should prioritize integrating objective frailty metrics and patient-reported outcomes to optimize VEN dosing. These methodological constraints highlight the need for prospective, randomized trials with comprehensive molecular profiling to validate the observed associations between treatment duration and clinical outcomes.

This study explored the efficacy, advantages, and safety of a short-term VA regimen in elderly patients with AML in China. Short-term VEN combined with AZA achieved a clinical remission rate similar to that of the long-term regimen, especially for patients aged ≥ 70 years with poor prognosis. It also reduced myelosuppression, shortened neutrophil recovery time, and reduced adverse events such as febrile neutropenia. Recent follow-up suggests that shortening VEN use may not affect survival time in elderly patients with AML.

## Acknowledgments

This is a short text to acknowledge the contributions of specific colleagues, institutions, or agencies that aided the efforts of the authors.

## Author contributions

**Conceptualization:** Shuangyue Li.

**Data curation:** Shuangyue Li, Yuxiao Wang.

**Formal analysis:** Zhuruohan Yu.

**Project administration:** Yuxiao Wang.

**Supervision:** Jiaojiao Yuan.

**Validation:** Ying Lu.

**Writing – review & editing:** Zhuruohan Yu, Renzhi Pei, Ying Lu.
